# Supplementary material for: Prevalence of hypertension and associated factors among adult residents in Arba Minch Health and Demographic Surveillance Site, Southern Ethiopia
Source: PLoS One. 2020 Aug 10;15(8):e0237333. doi: 10.1371/journal.pone.0237333 (PMC7416932; doi:10.1371/journal.pone.0237333)
Supplement: Attachment — Submitted filename: Response to Reviewers.odt [file pone.0237333.s002.odt]

Date: June 6, 2020
# Response to Reviewers
PONE-D-20-02687
Prevalence of Hypertension and Associated Factors among Adult Residents in Arba Minch Health and Demographic Surveillance Site.

Dear Editor 
I thank the editor and reviewers for the consideration and their constrictive comments. Here I included the response to your comment and uploaded the whole document. 
Regards, 
Befikadu Tariku
Summary 
The reviewer gave important comments. We modify based on the comment given. In addition, we attached document with trace changes and the revised. 
When submitting your revision, we need you to address these additional requirements.
1. Please ensure that your manuscript meets PLOS ONE's style requirements, including those for file naming. The PLOS ONE style templates can be found at
https://journals.plos.org/plosone/s/file?id=wjVg/PLOSOne_formatting_sample_main_body.pdf and
https://journals.plos.org/plosone/s/file?id=ba62/PLOSOne_formatting_sample_title_authors_affiliations.pdf
Response:- Corrected according to the guideline 
2. We note that you have stated that you will provide repository information for your data at acceptance. Should your manuscript be accepted for publication, we will hold it until you provide the relevant accession numbers or DOIs necessary to access your data. If you wish to make changes to your Data Availability statement, please describe these changes in your cover letter and we will update your Data Availability statement to reflect the information you provide.
Response:- We are decided to upload the dataset with the document 
3. Your ethics statement must appear in the Methods section of your manuscript. If your ethics statement is written in any section besides the Methods, please move it to the Methods section and delete it from any other section. Please also ensure that your ethics statement is included in your manuscript, as the ethics section of your online submission will not be published alongside your manuscript.
Response:- Correction made according to the suggestion 
4. We note you have included a table to which you do not refer in the text of your manuscript. Please ensure that you refer to Table 3 in your text; if accepted, production will need this reference to link the reader to the Table.
Response:- Correction made according to the suggestion 
# Review Comments to the Author
## Reviewer #1:
In the present manuscript the authors analyse the prevalence of arterial hypertension among adults residing in HDSS (Ethiopia). The prevalence was investigated in 3368 adults. The study has several limitations that must be addressed.
1. I suggest to use therm arterial hypertension instead of hypertension.
Response:- It is correct that we measured the arterial hypertension. But commonly description about hypertension related issues is about the arterial hypertension unless otherwise specified like pulmonary, portal etc. That is why we decided to use ‘hypertension’. 
2. I think Country (Ethiopia) may be mentioned in the tittle of manuscript and abstract.
Response:- We noted that and made the correction 
3. Perhaps hypertension is the leading risk factor for mortality, not leading risk...
Response:- Noted and made the correction 
4. In line 147: 44.8 (1107). Perhaps it should be 44.8 (11.07). Please check.
Response:- Noted and made the correction 
5. There are no p value in table 1 and 2.
Response:- On table 1 and 2, we only reported the descriptive information. That is why we do not include the p-value. Both the bivariate and multiple logistic regressions analysis is reported in table 3.
6. There are no abbreviations under table 2 and 3.
Response:- Noted and made the correction 
7. In line 174: it’s enough to write p=0.859.
Response:- Noted and made the correction 

## Reviewer #2:
This paper addresses a major public health concern across the globe and will make an important contribution to the literature in this research area. However, before this paper can be deemed publishable, authors would have to address the following key concerns provided by sections of the paper.

### Title:
Okay

### Abstract
This section is quite okay. However, authors should avoid reporting odds ratios as prevalence as stated in the last sentence of the Results sub-section. Also, authors should provide the AORs for the older age and the higher wealth index as they did for the other variables.
Response:- Based on the comment, we corrected the statements. 
### Introduction
While this section is quite okay, there are some few issues authors need to address.
The sentence in lines 42 and 43 needs to be revised as this is not clear; particularly “the leading cause of premature age-standardized mortality rates”.
Response:- Yes there was error and we corrected it. 
The 2001 statistics provided by the authors in lines 53-55 appear outdated. Authors should provide more recent figures, say 2015, as they did for the subsequent sentence in lines 55-56.
Response:- We used 2001 data-based report and know we corrected it. 
The sentence on line 60 needs to be revised for clarity and correctness.
Response:- Based on the comment, we corrected the statements. 
### Materials and Methods
In lines 66-71, while the authors did well by describing the geography of the study setting, it will be beneficial for them to also provide a few demographic descriptions, particularly the estimated population size of the study area.
Response:- Based on the suggestion, we included the population of the HDSS. 
In lines 86-88, it is not clear how the authors used Stata to randomly select participants from each stratum. Authors should be able to describe this process was done.
Response:- Arba Minch HDSS is a surveillance site where all the list of individuals in the site are registered with a unique identification (for both the individuals and house). So, we got this list and developed a sampling frame. Here we had 8 sampling frames (for each age group and sex categories). Using STATA software, we randomly selected the study subjects (individuals). 
Lines 104-105. This sentence should be revised into a better form.
Response:- Based on the suggestion, we corrected 
Authors did not describe all their variables of interest. It is more appropriate for authors to provide a separate sub-section to describe their outcome and predictor variables and their measurements. For convenience, authors should consider labelling BMI as underweight (<18.0kg/m2), normal (18.0–24.9kg/m2), and overweight (≥25kg/m2) instead of the real measurements.
Response:- Under Data collection instruments subsection of the methods, we described the method we followed for the measurement of the variable. And we indicated the methods we follow for generating new variables (based on the collected data) for analysis. Of course, some of the variable were missed and know we make the modification based on your recommendation. 
Authors stated that they used a p-value of 0.25 for the bivariate analysis but used 0.05 for the multivariate analysis. We know 0.25 is 75% while 0.055 is 95% CI. Authors should explain why they used these two different standards.
Response:- We did not decide whether there is association between outcome and predictors based on the p-value less than 0.25. This cut-off point (p-value less than 0.25) is used only to consider the predictor variable as a part for multivariate analysis. To define a variable (for both bivariate and multivariate analysis) as identified predictor for the outcome variable, we consider p-value less than 0.05 for both bivariate and multiple logistic regressions analysis.
Authors have stated that they have assessed multicollinearity using variable inflation factors. Authors should provide the resultant VIF value like they did for the Hosmer and Lemeshow goodness of fit test.
Response:- We corrected by including the maximum VIF value 

### Results
The presentation is generally okay but there are a few issues that need to be addressed.
In line 152, authors should rather say “more than one out of ten” instead of saying “nearly one out of ten”.
Response:- Based on the comment, we corrected it. 
In line 155, authors seem to want to say, “fresh food” instead of “flesh food”. Also, there appear to be no fresh food in Table 2 as stated by the authors.
Response:- We were explaining about meet consumption by says ‘flesh’. Now we changed to meet. 
Authors should avoid using prevalence in the multivariate analysis section as can be found in line 155.
Response:- We corrected throughout the document 
In line 182, authors should use “age groups” instead of “groups of age”.
Response:- Based on the comment, we corrected it. 
In line 184, authors should delete the “to” in the “concerning to”.
Response:- Based on the comment, we corrected it. 
Lines 187, 236, 277: Authors should avoid using “probability” and “prevalence” as they worked with only odds ratios.
Response:- We corrected throughout the document 
For easy visibility and convenience, authors should use asterisks (*) to indicate the significant values in Table 3.
Response:- Based on the comment, we corrected it. 
### Discussion
In line 203 and 204, revise the sentence. Use “have reported” instead of “were reported”.
Authors should revise the sentence in lines 207 and 208 as there is something wrong with it.
Response:- Based on the comment, we corrected it.
Line 209: Ethiopians instead of Ethiopian.
Response:- Based on the comment, we corrected it.
Lines 210, 214, 230, 231, 233, 239, 254: Avoid prevalence here. Odds, chances, and likelihood are examples author may use.
Response:- We corrected throughout the document 
Line 215: Iranians instead of Iranian.
Response:- Based on the comment, we corrected it.
Line 216, 217-218: Those who never married, not those never married.
Response:- Based on the comment, we corrected it.
Line 218 and others; Authors should explain the possible (speculative) reasons for their findings. For example, why would married women have low hypertension as found in your study? This should be done for the significant variables discussed in this section. Where this is not obvious, authors could briefly explain what could have led to this finding?
Response:- Based on the comment, we corrected it.
Line: 264: Reduced risk instead of reduced the risk.
Response:- Based on the comment, we corrected it.
Lines 266-267: Authors should either state and explain these factors or delete this statement as it appears incomplete and needless.
Response:- Based on the comment, we corrected it.
Lines 268-271: Authors should revise these sentences very well as they appear to be incomplete and unclear.
Response:- Based on the comment, we corrected it.
### Conclusion
Lines 273-275: Authors should adequately revise this sentence as it is unclear.
Response:- Based on the comment, we corrected it.
## General comments
While the paper has addressed an important public health issue and will contribute to the literature, it is noteworthy that the paper is also laden with some grammatical and diction challenges that have constrained the quality and flow of the paper. These are mainly found in the introduction and the discussion sections. Hence, this paper should be adequately proofread and revised accordingly before it be publishable.
