# Supplementary material for: Prevalence of hypertension and associated factors among adult residents in Arba Minch Health and Demographic Surveillance Site, Southern Ethiopia
Source: PLoS One. 2020 Aug 10;15(8):e0237333. doi: 10.1371/journal.pone.0237333 (PMC7416932; doi:10.1371/journal.pone.0237333)
Supplement: Attachment — Submitted filename: Response to Reviewers.odt [file pone.0237333.s003.odt]

PONE-D-20-02687R1
Prevalence of hypertension and associated factors among adult residents in Arba Minch Health and Demographic Surveillance Site, Southern Ethiopia.
# Journal Requirements:
Authors have effectively addressed the majority of the reviewer and editorial comments. However, there still remain a few important issues to be addressed.
Requirement 1: a) Authors byline and contributorship were not fully provided on the cover page (See cover page format). b) Authors level 2 headings do not abide by the “bold and font” format of the journal. c) The format style of the journal requires that authors provide unstructured abstract.
Response We corrected according to the Journals requirement 
Requirement 2: Authors have stated that they decided to upload the data set with the manuscript. However, this cannot be found.
Response We uploaded the minimum dataset. 

# Reviewers' comments and evaluation:
## Reviewer 1
1. Comment 3: Authors did not address this comment as they stated (see Line 57 of track changes) and should be addressed.
Response: Thank you for the comment. We noted that and corrected it. 
## Reviewer 2
Introduction
2. Little has been done by authors to make the statement: “the leading cause of premature age-standardized mortality rates” (lines 42 and 43 of original version) meaningfully clear. Authors should recast this statement to make it clearer.
Response: Thank you for the comments. Yes, we noted that and correct 
3. Authors have addressed the comment of the reviewer (originally line 60); however, the second statement still appears incomplete (Lines 66-68) and should be revised to make it complete. 
Response: Yes, we noted that and correct 
Materials and Methods
4. Authors have provided the recommended information regarding lines 66-71, but have to provide citation for such report.
Response: We get from the database of the Arba Minch HDSS. We got the data while during the sampling frame. But we also included the district’s projected population of 2017, which have also reference. 
5. Authors provided a cogent explanation to the comment regarding the sampling procedure (Lines 86-88) but authors should incorporate it in the manuscript for readers to clearly understand.
Response: Noted and made some correction. 
6. Authors did not fully address this comment regarding the description of the variable of interest. Also, the BMI categories such as underweight, normal and overweight appear only in the methods section but did not reflect in the tables and the analysis. These should be addressed by the authors
Response: Regarding BMI cut-off points, the data were categorized with the cutoff of this one (18.5). The sub-title is incorporated.  
Discussion
7. The second comment (lines 207-208) has not been addressed as stated by the authors in their response  letter.  Authors are invited to address this. 
Response: - Based on the comment, we corrected it.

General comments
8. Authors did not respond to the comment as to whether they have proofread and revised the manuscript to make it publishable. I invite authors  to address this issue.
Response: - Yes, we read the whole documents and corrected identified errors. 
The reviewer gave important comments. We modify based on the comment given. In addition, we attached document with trace changes and the revised.
