# Supplementary material for: Prevalence of hypertension and associated factors among adult residents in Arba Minch Health and Demographic Surveillance Site, Southern Ethiopia
Source: PLoS One. 2020 Aug 10;15(8):e0237333. doi: 10.1371/journal.pone.0237333 (PMC7416932; doi:10.1371/journal.pone.0237333)
Supplement: Attachment — Submitted filename: Response to Reviewers.odt [file pone.0237333.s004.odt]

PONE-D-20-02687R1
Prevalence of hypertension and associated factors among adult residents in Arba Minch Health and Demographic Surveillance Site, Southern Ethiopia.

Editor comments:
Authors have effectively addressed virtually all the comments. However, a few important issues remain to be addressed.
Introduction
Sentence in Lines 65-66 appears to be a repetition of that in Lines 50-51. Authors should delete sentence in lines 65-66: In Ethiopia, hypertension was one of the leading causes of age standardized death rates in 2015.
Response Done according to the recommendation 
Tables
Authors should replace the figures with the BMI categories such as underweight, normal and overweight as provided in the methods and the results sections to ensure consistency and easy reference.
Response Done according to the recommendation
Discussion
Lines 257: Ethiopia, not Ethiopian.
Response Done according to the recommendation
Lines 287-289 should read: On further analysis, the relationship is consistent with that of some high-income countries (countries from USA/Canada and Europe) but inverse for some African countries.
Response Done according to the recommendation 

Thank you for your comment. We modify based on the comment given. In addition, we attached document with trace changes and the revised.
